# Impact of the Gastric Acid Suppressant Use on the Safety and Effectiveness of EGFR-TKIs: A Systematic Review and Meta-Analysis

**DOI:** 10.3389/fphar.2022.796538

**Published:** 2022-06-20

**Authors:** Xin Du, Wei Liu, Ken Chen, Ziyu Wang, Xinyi Li, Li Yang, Xiaohui Xie

**Affiliations:** ^1^ School of Pharmaceutical Sciences, Peking University, Beijing, China; ^2^ Department of Pharmacy, Peking University Third Hospital, Beijing, China; ^3^ College of Pharmacy, University of Nebraska Medical Center, Omaha, NE, United States; ^4^ School of Basic Medical Sciences and Clinical Pharmacy, China Pharmaceutical University, Nanjing, China

**Keywords:** EGFR-TKIs, GASs, drug–drug interaction, effectiveness, safety, NSCLC

## Abstract

**Background** The use of gastric acid suppressants (GASs) has an influence on the exposure of some epidermal growth factor receptor-tyrosine kinase inhibitors (EGFR-TKIs) and therefore may affect the effectiveness and safety of EGFR-TKIs. The impact of GASs, including proton pump inhibitors (PPIs) and histamine type 2 receptor antagonists (H_2_RAs), on the effectiveness and safety of EGFR-TKIs remains unclear. We conducted a meta-analysis to explore the impact of GASs on the effectiveness and safety of EGFR-TKIs in non–small cell lung cancer (NSCLC) patients.

**Method** We searched the PubMed, Embase, Cochrane Library, China National Knowledge Infrastructure, and Wanfang databases thoroughly from inception to 2nd February 2021, including the studies for NSCLC patients who used GASs, offering the adjusted hazard ratio (HR) of effectiveness outcomes such as overall survival (OS) and progression-free survival (PFS) or adjusted odds ratio (OR) of the adverse drug reaction (ADRs), and the results were calculated with a random effect. Two researchers independently screened the literature, extracted data, and evaluated the quality. Stata 15.0 was used for meta-analysis.

**Result** Twelve studies were finally included. Nine of them were cohort studies, and three of them were case–control studies. For effectiveness outcomes, the use of GASs was associated with shorter PFS (HR 1.66 [1.40, 1.98]) and OS (HR 1.50 [1.31, 1.72]), and the use of PPIs was associated with shorter OS (HR 1.56 [1.21, 2.02]), regardless of the overlap time and type of EGFR-TKIs. For safety outcomes, the use of GASs (OR 1.98 [1.19, 3.31]) or PPIs (OR 1.91 [1.17, 3.12]) were both associated with an increased risk of hepatotoxicity.

**Conclusion** The concomitant use of GASs is associated with shorter PFS and OS for NSCLC patients taking EGFR-TKIs and is also associated with a higher risk of hepatotoxicity. The co-administration of GASs should be avoided; if they cannot be avoided, H_2_RAs is a better choice.

**Systematic Review Registration:** (https://www.crd.york.ac.uk/prospero/display_record.php?ID=CRD42021235018), identifier (PROSPERO 2021 CRD42021235018)

## Introduction

Lung cancer is the leading cause of death in patients with malignant tumors worldwide and non–small cell lung cancer (NSCLC) accounts for approximately 85% of all lung cancers (Parkin et al., 2001). Epidermal growth factor receptor (EGFR) mutations present in 10–50% of patients with stage IV NSCLC are most prevalent in women, never-smokers, adenocarcinomas, and East Asian patients (Mitsudomi and Yatabe, 2007; Rosell et al., 2009). Tyrosine kinase inhibitors (TKIs) targeting the EGFR (e.g., gefitinib, erlotinib, afatinib, and osimertinib) are currently indicated as the first-line treatment of sensitizing EGFR mutation-positive stage IV NSCLC (Indini et al., 2020).

The histamine type 2 receptor antagonists (H_2_RAs) and proton pump inhibitors (PPIs) are the major gastric acid suppressants (GASs) that are widely used for many indications requiring acid suppression, such as peptic ulcer disease and upper gastrointestinal bleeding. Compared to H_2_RAs, PPIs have shown a stronger acid inhibitory effect by blocking H^+^/K^+^ ATPase (Ahmed and Clarke, 2021) on the parietal cells. One study showed that H_2_RAs could suppress 70% of the mean 24-h acidity after taking them for 2 weeks, while PPIs showed over 90% (Howden and Hunt, 1990).

The co-administration of GASs such as PPIs and H_2_RAs is reported in 33.2–46.3% of the lung cancer patients (Smelick et al., 2013; Saito et al., 2020). Another study showed that 21.7% of elder lung cancer patients concomitantly use PPIs and erlotinib (Sharma et al., 2019). Since the solubility of some EGFR-TKIs is pH-dependent, the absorption of EGFR-TKIs may be influenced by GASs through altering gastric pH. Preclinical studies showed that gefitinib’s solubility could decrease from 21 mg/ml to <0.001 mg/ml when gastric pH increases from 1 to 7. For erlotinib, its maximal solubility achieves at pH = 4 (Budha et al., 2012). Clinical studies showed that the use of GASs, especially PPIs, can decrease the exposure of gefitinib, erlotinib, and dacomitinib significantly (Kletzl et al., 2015; Ruiz-Garcia et al., 2016; Yokota et al., 2017; Yasumuro et al., 2018). The effect appears to be more robust and durable for PPIs compared with H_2_RAs (Kletzl et al., 2015; Yokota et al., 2017). The concomitant use of gefitinib and PPIs could result in a sustainable decrease of gefitinib concentration, while the effect time was shortened to 6–24 h, when used together with H_2_RAs (Yokota et al., 2017).

To manage this drug–drug interaction, the package inserts of some EGFR-TKIs recommend separate administration, especially for the H_2_RAs (FDA, 2004; FDA, 2015; FDA, 2018). The recommendations were primarily based on PK data rather than clinical endpoints. Therefore, it is necessary to explore the influence of GASs on the effectiveness and safety of EGFR-TKIs to provide more information on current recommendations.

## Materials and Methods

### Literature Search

We searched the PubMed, Embase, Cochrane Library, China National Knowledge Infrastructure, and Wanfang databases. Our search strategy included ((omeprazole or dexlansoprazole or lansoprazole or esomeprazole or pantoprazole or rabeprazole or ilaprazole or “proton pump inhibitor” or PPI or PPIs) or (ranitidine or cimetidine or famotidine or nizatidine or “H_2_ receptor inhibitor” or H_2_RA or “histamine 2 receptor antagonist” or H_2_RAs) or “gastric acid suppressant”) and (gefitinib or erlotinib or afatinib or icotinib or osimertinib or dacomitinib or IRESSA or TARCEVA or GILOTRIF or Conmana or TAGRISSO or VIZIMPRO or EGFR-TKI or EGFR-TKIs). We did not apply any restrictions on publication date, language, or publication status to the searches.

### Study Eligibility and Selection

We included studies that 1) recruited NSCLC patients who used EGFR-TKIs; 2) compared the outcomes of patients who used GASs with those who did not; 3) reported the adjusted effectiveness or safety outcomes of EGFR-TKIs such as PFS, OS, and ADR; and 4) were randomized controlled trials (RCTs), cohort studies, or case–control studies. Our exclusion criteria were 1) studies published as an abstract in proceedings of scientific events; 2) studies not in English or Chinese.

Two reviewers (XD and WL) independently screened titles and abstracts and reviewed the full texts of potential studies to determine eligibility. Disagreements were resolved by discussion or by a third reviewer (KC). If an article was not included, the reason was recorded.

### Data Collection and the Assessment of the Risk of Bias

Two reviewers (XD and WL) independently extracted data including author names, publication year, study design, the definition of using GASs, sample size, and outcomes. Effectiveness outcomes were mainly PFS and OS. PFS was calculated from the date of initial treatment with EGFR-TKIs until disease progression, clinical deterioration, the date of last contact, or death. OS was calculated from the date of initial treatment with EGFR-TKIs until death. The PFS and OS were measured at the end of the follow-up. Safety outcomes were measured at the end of the follow-up too. Adjusted HR and OR were extracted for effectiveness and safety outcomes, respectively. The data were extracted and summarized into an Excel worksheet.

Two reviewers (ZW and XL) assessed the risk of bias independently using a modified version of the Cochrane criteria for RCTs, a modified version of the Newcastle–Ottawa instrument for cohort studies and an instrument developed specifically for case–control studies. Reviewers judged each criterion as definitely or probably low or high risk of bias and resolved any disagreements by discussion or, if necessary, by consultation with a third reviewer (KC).

### Statistical Analysis

We analyzed the results of patients who used the GASs, PPIs, or H_2_RAs, separately. To account for the expected heterogeneity among studies, the DerSimonian and Laird random-effect model was used for the analysis of adjusted HRs and ORs, and the corresponding forest plots were drawn. The *I* ^*2*^ (%) was used to assess the heterogeneity of the selected research studies. The subgroup analysis was performed according to the overlap time of GASs and EGFR-TKIs use, and sensitivity analysis was conducted by eliminating studies one by one and reanalysis. Data analysis was performed by Stata 15.0 software.

## Results

### Study Eligibility and Assessment of the Risk of Bias

**FIGURE 1:**
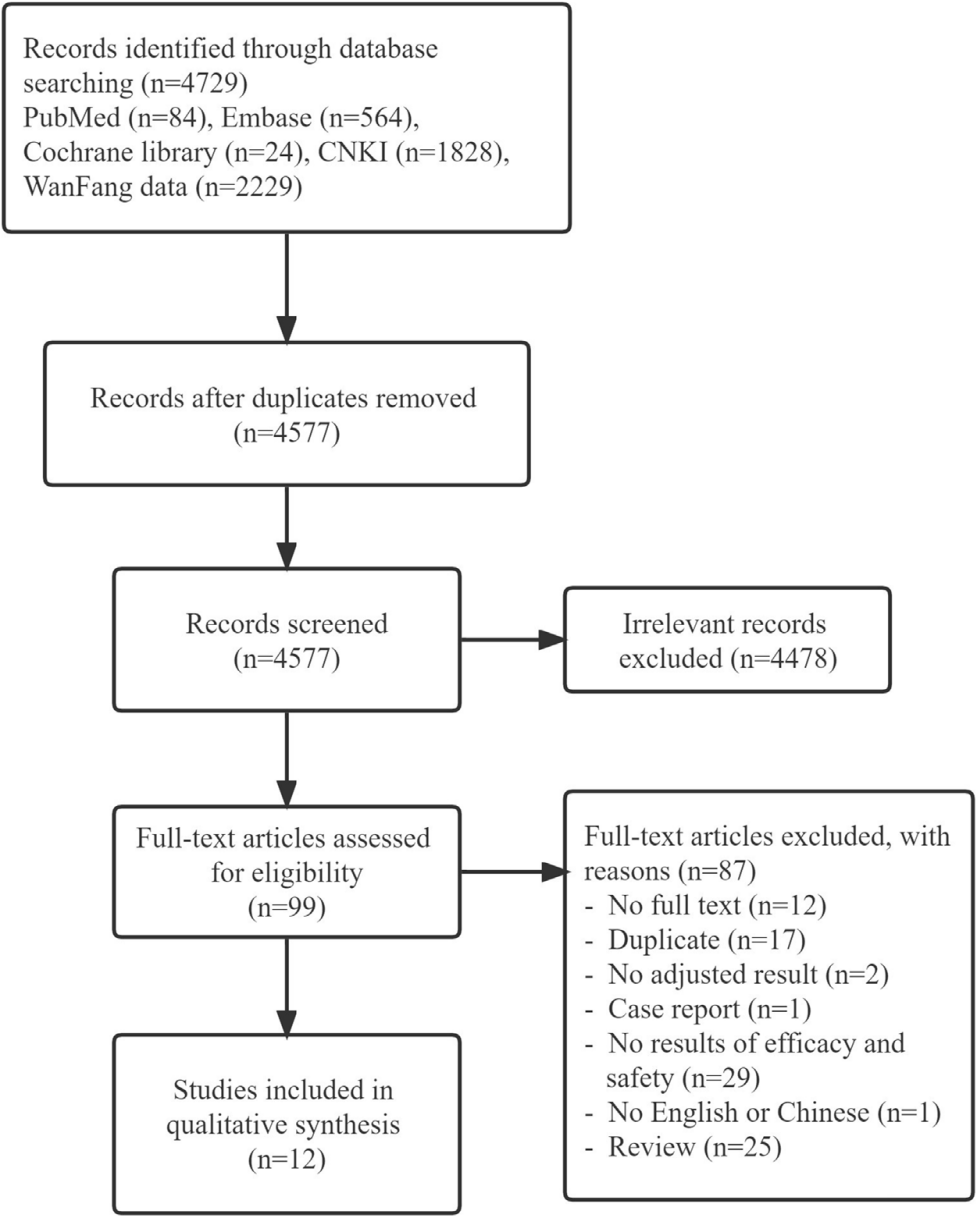
Flow chart for the determination of included studies.

The literature screening process and results are shown in Figure 1. A total of 4,729 related literature were retrieved. After tiered screening, a total of 12 studies met eligibility criteria, including nine cohort and three case–control studies. Nine of 12 studies reported effectiveness outcomes and three studies reported safety outcomes. The quality evaluation showed that all cohort studies and one case–control study were of high quality, while the other two case control studies were of low quality. The main characteristics of the included studies are presented in Table 1, and the quality evaluation is shown in Supplementary Table S1,S2.

**TABLE 1:** Main characteristics of the included studies.

Reference	Study design	Type of EGFR-TKI	No. of participant	Exposure (PPI%)	Comparison	Definition of GAS (overlap time)	Outcome
(Chen et al., 2016)	Cohort	First-generation	269	GASs (31.58)	No GASs	>30%	OS
(Chu et al., 2015)	Cohort	Erlotinib	507	GASs (93)	No GASs	≥20%	PFS and OS
(Fang et al., 2019)	Cohort	Gefitinib	1,133	PPIs (100)	No PPIs	0–20%	OS
			1,114			>20%	OS
(Guo et al., 2020)	Cohort	Gefitinib	188	GASs (93.88)	No GASs	Use	PFS
						50%	PFS
(Hilton et al., 2013)	Cohort	Erlotinib	485	GASs (NA)	No GASs	Use	PFS and OS
(Kumarakulasinghe et al., 2016)	Cohort	Gefitinib/erlotinib	157	GASs (NA)	No GASs	≥30%	PFS and OS
(Nieves Sedano et al., 2018)	Cohort	Gefitinib/erlotinib	163	GASs (72.03)	No GASs	≥20%	PFS
(Sharma et al., 2019)	Cohort	Erlotinib	5,496	PPIs (100)	No PPIs	≥30% in the first 3M	Death in 90d/1y
(Zenke et al., 2016)	Cohort	Gefitinib/erlotinib	130	GASs (57.4)	No GASs	Use	PFS and OS
(Cho et al., 2018)	Case–control	Gefitinib	374	GASs (31.50)	No GASs	NA	Hepatotoxicity
(Han et al., 2020)	Case–control	Gefitinib/erlotinib	459^a^	GASs (NA)	No GASs	NA	Hepatotoxicity
(Kim et al., 2018)	Case–control	Erlotinib	155	GASs (45.71)	No GASs	NA	Hepatotoxicity

a Participant number was calculated by the number of EGFR-mutant patients in Cho et al. (2018) and Kim et al. (2018)’s studies.

### Influence on the Effectiveness of EGFR-TKIs

**FIGURE 2:**
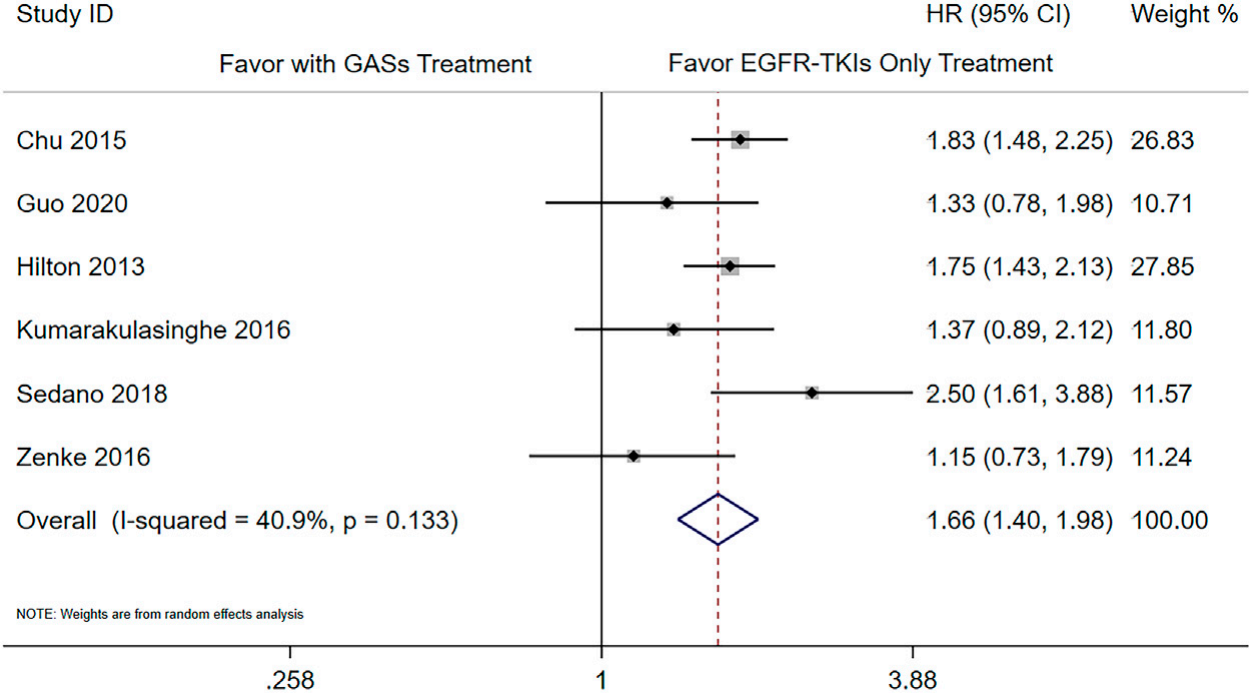
Influence of the concomitant use of GASs on the PFS.

**FIGURE 3:**
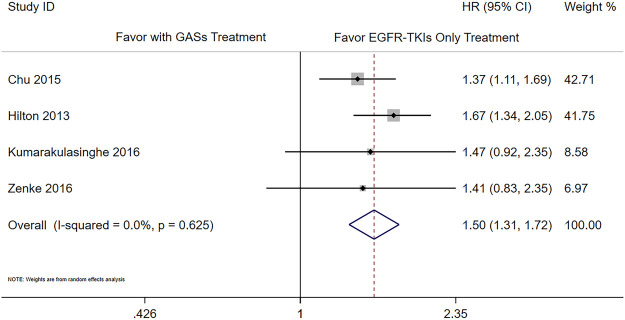
Influence of the concomitant use of GASs on the OS.

There were six studies that reported the adjusted results of effectiveness outcomes using GASs (Chu et al., 2015; Guo et al., 2020; Hilton et al., 2013; Kumarakulasinghe et al., 2016; Nieves Sedano et al., 2018; Zenke et al., 2016). The meta-analysis results showed that the use of GASs was associated with shorter PFS (HR 1.66 [1.40, 1.98], Figure 2) and OS (HR 1.50 [1.31, 1.72], Figure 3). Three studies focused on PPIs (Sharma et al., 2019; Chen et al., 2016; Fang et al., 2019); two of them reported PFS and OS (Chen et al., 2016; Fang et al., 2019), and the other reported death rate in 90 days and 1 year (Sharma et al., 2019). The meta-analysis of the two studies (Chen et al., 2016; Fang et al., 2019) showed that the use of PPIs was associated with shorter OS (HR 1.56 [1.21, 2.02], Supplementary Figure S1). The other study reported that the use of PPIs was associated with a higher death rate in 90 days (Sharma et al., 2019) (adjusted HR 1.21, [1.07, 1.38]) and in 1 year (adjusted HR 1.11, [1.02, 1.20]). One of the aforementioned studies presented that the use of H_2_RAs did not influence the OS (Chen et al., 2016) (adjusted HR 1.46, [0.92, 2.33]).

**FIGURE 4:**
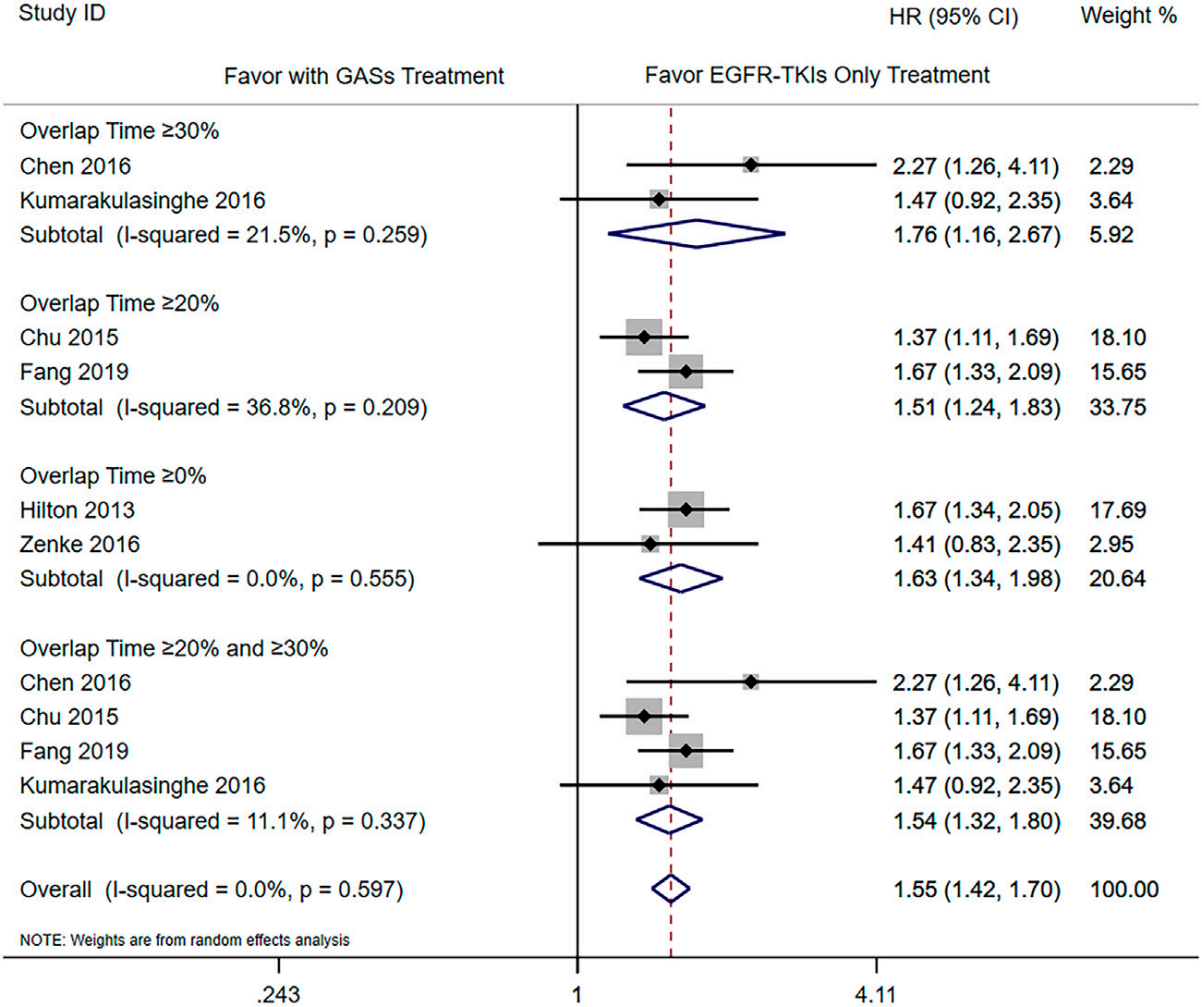
Influence of concomitant use of GASs in different overlap time on the OS.

**FIGURE 5:**
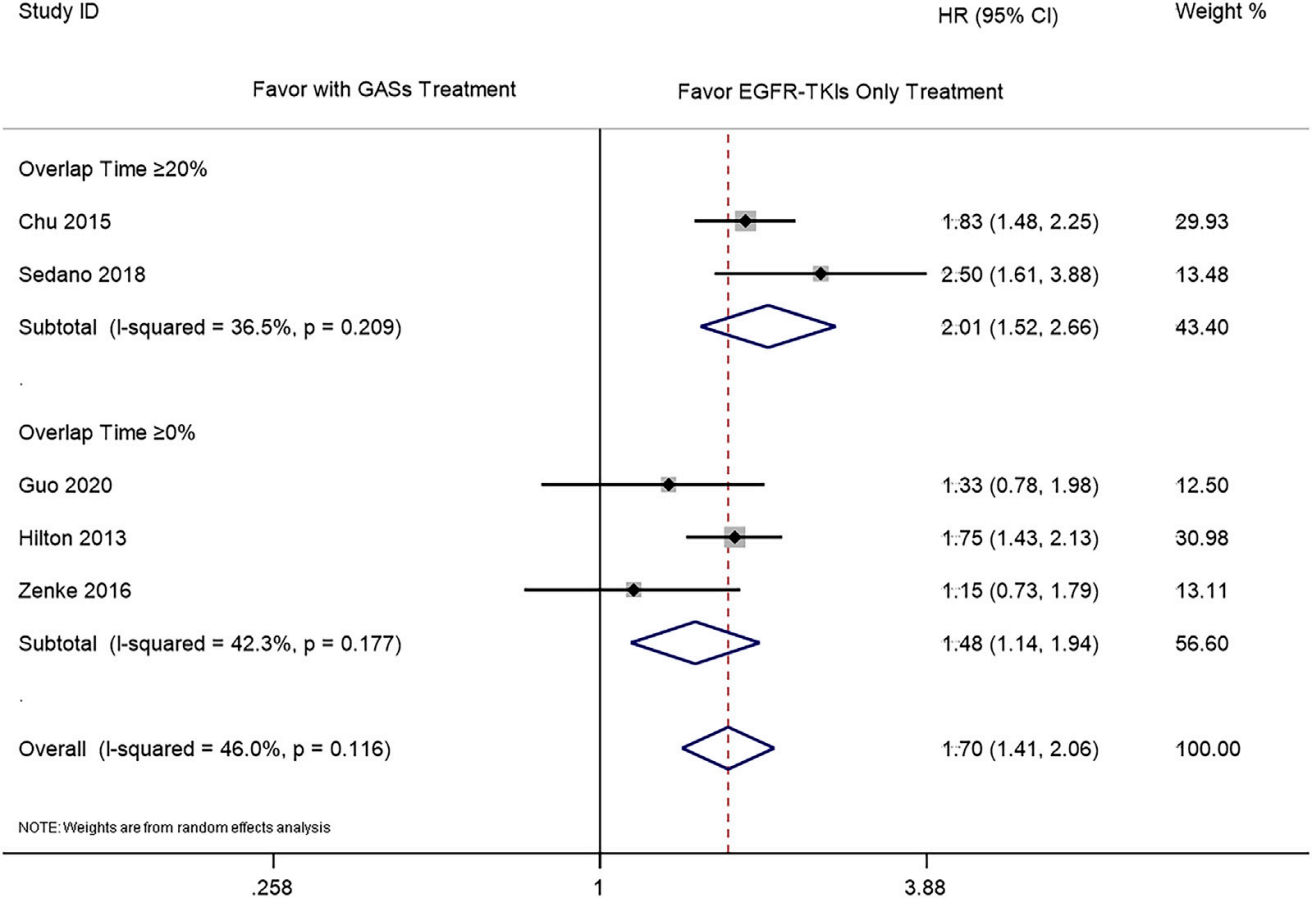
Influence of concomitant use of GASs in different overlap time on the PFS.

The subgroup analysis was carried out according to the different overlap times of GASs and EGFR-TKIs. Since the previous result showed that the use of H_2_RAs did not influence the OS, we only combined the results of PPIs and GASs. The included studies used different overlap times such as ≥0% (Hilton et al., 2013; Zenke et al., 2016; Guo et al., 2020), 0–20% (Guo et al., 2020), ≥20% (Chu et al., 2015; Nieves Sedano et al., 2018; Fang et al., 2019), ≥30% (Chen et al., 2016; Kumarakulasinghe et al., 2016), and ≥50% (Guo et al., 2020), respectively. For the OS, different overlap times such as ≥30% (HR 1.76 [1.16, 2.67]), ≥20% (HR 1.51 [1.24, 1.83]), ≥0% (HR 1.63 [1.34, 1.98]), and ≥20% and ≥30% (HR 1.54 [1.32, 1.80]) were all associated with a shorter OS (Figure 4). For PFS, the results showed that the overlap time of both ≥0% (HR 1.48 [1.14, 1.94]) and ≥20% (HR 2.01 [1.52, 2.66]) were associated with a shorter PFS; the higher overlap time showed a greater influence (Figure 5).

### Influence on the Safety of EGFR-TKIs

For GASs, three studies reported adjusted safety results (Cho et al., 2018; Kim et al., 2018; Han et al., 2020). Two of them (Cho et al., 2018; Kim et al., 2018) reported all-grade hepatotoxicity, while the other (Han et al., 2020) analyzed grade 3–4 hepatotoxicity whose data came from the former two studies. The results showed that the use of GASs was associated with a higher risk of all-grade hepatotoxicity (OR 1.98 [1.19, 3.31], Supplementary Figure S2) as well as grade 3–4 hepatotoxicity (adjusted OR 3.757 [1.642, 8.601]). The use of PPIs was associated with both all-grade hepatotoxicity (OR 1.91 [1.17, 3.12], Supplementary Figure S3) and grade 3–4 hepatotoxicity (adjusted OR 3.365 [1.376, 8.227]).

For H_2_RAs, there were two studies (Cho et al., 2018; Han et al., 2020) that reported all-grade hepatotoxicity and grade 3–4 hepatotoxicity, respectively. The results showed that the use of H_2_RAs was associated with all-grade hepatotoxicity (OR 1.566, [1.044, 2.348]) and grade 3–4 hepatotoxicity (adjusted OR 2.823, [1.279, 6.232]).

### Sensitivity

Sensitivity analysis showed that the impact of using GASs on PFS and OS was stable, while for the use of PPIs, it had no significant influence on the OS when the result of Fang (overlap time >20%) was excluded.

## Discussion

This is the first meta-analysis exploring the impact of PPIs and H_2_RAs on both effectiveness and safety of EGFR-TKIs using the adjusted results and also explored the impact according to different overlap times. According to the results, the use of GASs was found to be associated with decreased OS and PFS, even with an overlap of co-administration time shorter than 20%. In addition, a higher risk of all-grade hepatotoxicity was observed regardless of the use of GASs, PPIs or H_2_RAs.

A previous meta-analysis (Indini et al., 2020) focused on multiple cancer types, which only included five studies for NSCLC. Our study included 12 studies for NSCLC patients reporting adjusted outcomes. The reason why we restricted to adjusted data is that the survival time of cancer patients was influenced by numerous factors, which should be appropriately accounted for during statistical analysis to reduce possible confounding effects.

Different overlap times may affect the result. Guo et al. (2020) used a lower overlap time and showed an insignificant impact with overlap time ≥0% (HR 1.33, [0.784, 1.98]), and the impact turned to be significant when the overlap time was ≥50% (HR 0.067, [0.033, 0.136]).

There may be several reasons that H_2_RAs did not influence the OS. First, there is only one study reporting the influence of H_2_RAs on OS with inadequate power to detect the significance. Second, as mentioned earlier, H_2_RAs have a shorter effect on acid suppression compared to PPIs. Third, some H_2_RAs such as ranitidine and cimetidine are CYP3A inhibitors, while most of EGFR-TKIs are metabolized by CYP3A4 (Hakkola et al., 2020). Therefore, the use of H_2_RAs could increase the EGFR-TKI concentration which may offset some of the influence from the altered pH.

A higher risk of all-grade hepatotoxicity was observed in patients using any kind of GASs. It might be due to the interaction of transporters and enzymes. Nearly all of the EGFR-TKIs are substrates for P-glycoprotein (P-gp) and breast cancer resistance protein (BCRP), while the PPIs are P-gp and BCRP inhibitors. Moreover, the therapeutic concentration of some PPIs such as omeprazole and pantoprazole could be higher than their half-maximal inhibitory concentrations (IC_50_) of P-gp and BCRP (Stedman and Barclay, 2000; Pauli-Magnus et al., 2001; Suzuki et al., 2009). For omeprazole, the IC_50_ values of P-gp and BCRP are 17.7 and 17.6 μM, separately, while its C_max_ could reach 8 μg/ml (23.2 μM). For pantoprazole, the IC_50_ of BCRP is 5.5 μM, and its C_max_ could reach 3.3 μg/ml (8.62 μM). This may result in the increased liver concentration of EGFR-TKIs, although this effect may not be huge enough to offset the decreased absorption (we still see the decreased plasma concentration), but we speculated the increased local concentration may result in hepatotoxicity.

According to the results, we suggested the co-administration of EGFR-TKIs and GASs should be avoided if possible, even with intake–separation. Kwok et al. (2020) carried out a study and showed that even administrating GASs and EGFR-TKIs with a separation of 12 h could still lead to a shorter PFS and a shorter OS in patients using gefitinib. Therefore, the antacids may be a better choice because of the relatively short duration of anti-acid effects that could be easily separated from the EGFR-TKIs usage. If the use of H_2_RAs or PPIs cannot be avoided, the H_2_RAs may be the choice compared with the PPIs, providing strict monitoring of the liver function.

This meta-analysis has limitations. Our data was limited; therefore, some of the subgroup analyses such as comparing the different influence of PPIs and H_2_RAs and different overlap times were not feasible. In addition, the included studies were all about gefitinib or erlotinib. We will have to wait for more studies of the newer EGFR-TKIs. Third, both PPIs and H_2_RAs are available as over-the-counter medications in some countries, and there may be some usage that is not recorded that could influence the results of the original studies and also our results.

## Conclusion

The use of GASs, especially PPIs, could influence the effectiveness and safety of EGFR-TKIs. The PFS and OS of NSCLC patients taking EGFR-TKIs are shorter when co-administered with GASs. When the overlap time exceeds 20%, the negative effect of GASs on OS becomes greater. The concomitant use of GASs is associated with a higher risk of all-grade hepatotoxicity. The co-administration of GASs and EGFR-TKIs should be avoided. If it cannot be avoided, H_2_RAs may be a better choice, with close monitoring of the liver function during the co-administration, especially for gefitinib or erlotinib. However, the results still have some limitations, and further studies with improved designs are necessary to confirm these results.

## Data Availability

The original contributions presented in the study are included in the article/Supplementary Material, further inquiries can be directed to the corresponding authors.

## Abbreviations

NSCLC, non–small cell lung cancer; EGFR-TKIs, epidermal growth factor receptor tyrosine kinase knhibitors; GASs, gastric acid suppressants; PPIs, proton pump inhibitors; H_2_RAs, histamine type 2 receptor antagonists; EGFR, epidermal growth factor receptor; TKIs, tyrosine kinase inhibitors; PFS, progression-free survival; OS, overall survival; ADRs, adverse drug reactions; RCTs, randomized controlled trials; HR, hazard ratio; OR, odds ratio; P-gp, P-glycoprotein; BCRP, breast cancer resistance protein; IC_50_, half-maximal inhibitory concentrations.
