# Supplementary material for: Impact of the Gastric Acid Suppressant Use on the Safety and Effectiveness of EGFR-TKIs: A Systematic Review and Meta-Analysis
Source: Front Pharmacol. 2022 Jun 20;13:796538. doi: 10.3389/fphar.2022.796538 (PMC9252455; doi:10.3389/fphar.2022.796538)
Supplement: Supplementary file 1 [file DataSheet1.pdf]

Table 1 Risk of bias in Cohort Studies using Newcastle-Ottawa scale

Study	From the same population	Assessment of exposure	Outcome not present at start	Comparability	Assessment of prognostic factors	Assessment of outcome	Adequate follow-up	Co-Interventions similar
Chen 2016[19]	Yes	Yes	Yes	Probably yes	Yes	Probably yes	Probably yes	Probably yes
Chu 2015[20]	Yes	Yes	Probably yes	Probably yes	Yes	Probably yes	Yes	Probably yes
Fang 2019[21]	Yes	Yes	Yes	Probably yes	Yes	Probably yes	Yes	Probably yes
Guo 2020[22]	Yes	Yes	Probably yes	Probably yes	Yes	Probably yes	Yes	Probably yes
Hilton 2013[23]	Yes	Yes	Probably yes	Probably yes	Yes	Probably yes	Yes	Probably yes
Kumarakulasinghe 2016[24]	Yes	Yes	Yes	Probably yes	Yes	Probably yes	Yes	Probably yes
Sedano 2018[25]	Yes	Yes	Probably yes	Probably yes	Yes	Probably yes	Yes	Probably yes
Sharma 2019[9]	Probably yes	Yes	Yes	Probably yes	Yes	Probably yes	Yes	Probably yes
Zenke 2016[26]	Yes	Yes	Probably yes	Probably yes	Yes	Probably yes	Yes	Probably yes

Table 2 Risk of bias in Case Control Studies using Newcastle-Ottawa scale

Study	Assessment of exposure	Confident outcome	Cases properly selected	Controls properly selected	Match
Cho 2018[27]	Yes	probably no	Yes	Yes	Probably yes
Han 2020[28]	Yes	probably no	Yes	Yes	Probably yes
Kim 2018[29]	Yes	Yes	Yes	Yes	Probably yes

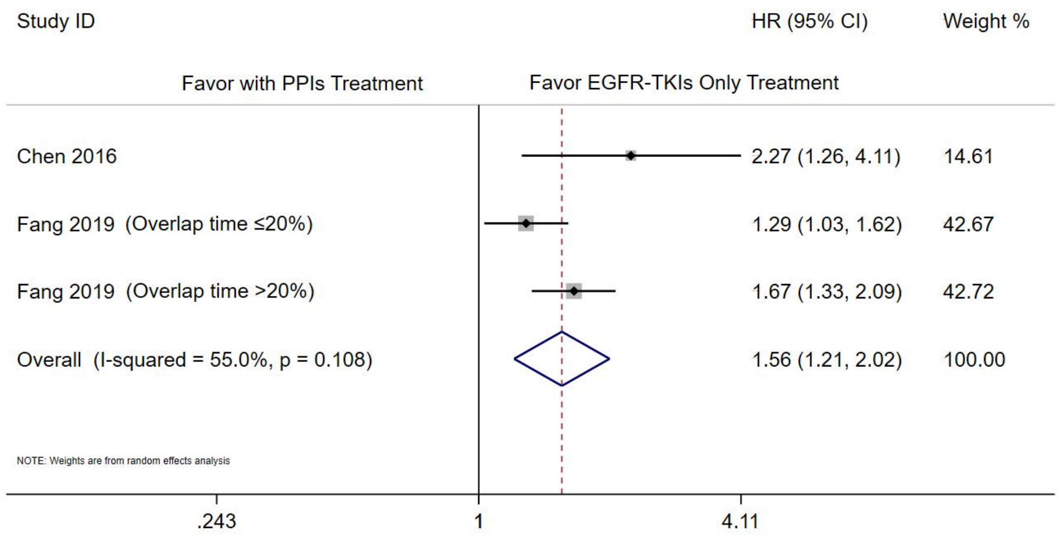

Figure 1 Influence of the concomitant use of PPIs on the OS

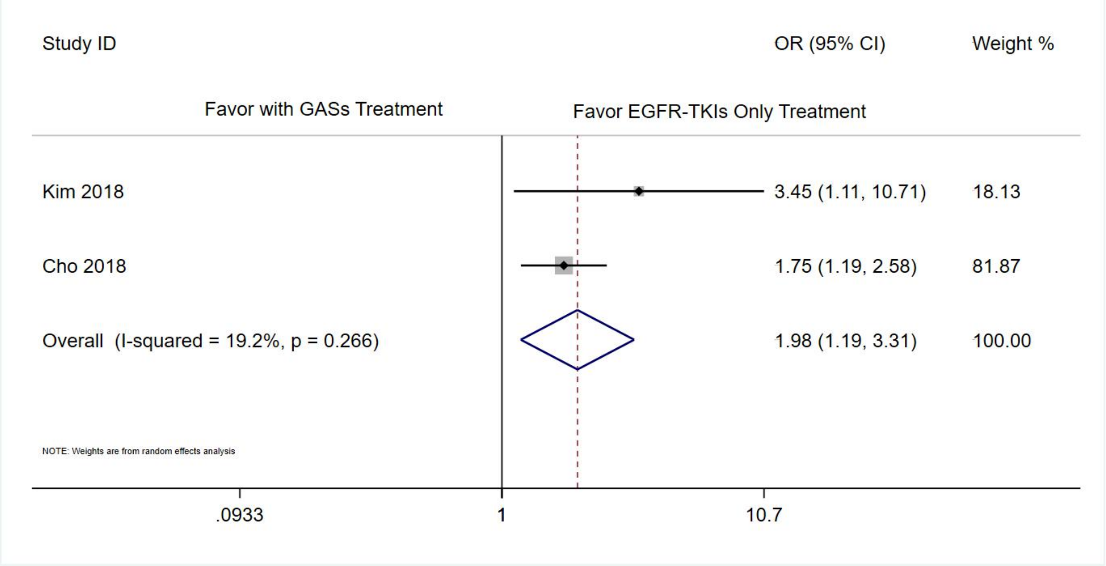

Figure 2 Influence of the use of GASs on the hepatotoxicity

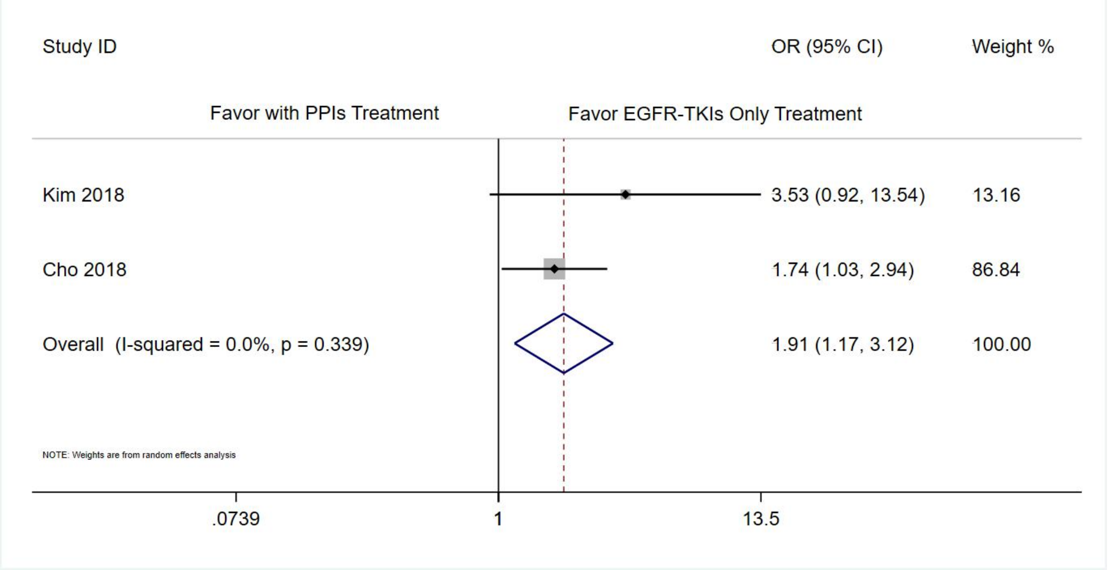

Figure 3 Influence of the use of PPIs on the hepatotoxicity

[9]. Sharma M, Holmes HM, Mehta HB, et al. The concomitant use of tyrosine kinase inhibitors and proton pump inhibitors: Prevalence, predictors, and impact on survival and discontinuation of therapy in older adults with cancer[J]. Cancer, 2019, 125(7):1155-62.

[19]. Chen YM, Lai CH, Chang HC, et al. Antacid Use and De Novo Brain Metastases in Patients with Epidermal Growth Factor Receptor-Mutant Non-Small Cell Lung Cancer Who Were Treated Using First-Line First-Generation Epidermal Growth Factor Receptor Tyrosine Kinase Inhibitors[J]. PLoS One, 2016, 11(2):e0149722.

[20]. Chu MP, Ghosh S, Chambers CR, et al. Gastric Acid suppression is associated with decreased erlotinib efficacy in non-small-cell lung cancer[J]. Clin Lung Cancer, 2015, 16(1):33-9.

[21]. Fang YH, Yang YH, Hsieh MJ, et al. Concurrent proton-pump inhibitors increase risk of death for lung cancer patients receiving 1st-line gefitinib treatment - a nationwide population-based study[J]. Cancer Manag Res, 2019, 11:8539-46.

[22]. Guo Z, Du Q, Ye X, et al. Concomitant administration of gastric acid suppression might attenuates the clinical efficacy of gefitinib: A single cancer center retrospective study[J]. Journal of Chinese Pharmaceutical Sciences, 2020, 29(3):192-98.

[23]. Hilton JF, Tu D, Seymour L, et al. An evaluation of the possible interaction of gastric acid suppressing medication and the EGFR tyrosine kinase inhibitor erlotinib[J]. Lung Cancer, 2013, 82(1):136-42.

[24]. Kumarakulasinghe NB, Syn N, Soon YY, et al. EGFR kinase inhibitors and gastric acid suppressants in EGFR-mutant NSCLC: a retrospective database analysis of potential drug interaction[J]. Oncotarget, 2016, 7(51):85542-50.

[25]. Sedano MN, Teller JMC, Muñoz CG, et al. Clinical impact of gastric acid suppressing medication on the effectiveness of tyrosine kinase inhibitors in lung cancer patients[J]. Journal of BUON, 2018, 23(3):647-53.

[26]. Zenke Y, Yoh K, Matsumoto S, et al. Clinical Impact of Gastric Acid-Suppressing Medication Use on the Efficacy of Erlotinib and Gefitinib in Patients With Advanced Non-Small-Cell Lung Cancer Harboring EGFR Mutations[J]. Clin Lung Cancer, 2016, 17(5):412-18.

[27]. Cho S, Yee J, Kim JY, et al. Effects of Concomitant Medication Use on Gefitinib-Induced Hepatotoxicity[J]. J Clin Pharmacol, 2018, 58(2):263-68.
[28]. Han JM, Han HW, Yee J, et al. Factors affecting high-grade hepatotoxicity of tyrosine kinase inhibitors in cancer patients: a multi-center observational study[J]. Eur J Clin Pharmacol, 2020, 76(8):1183-91.
[29]. Kim MK, Yee J, Cho YS, et al. Risk factors for erlotinib-induced hepatotoxicity: a retrospective follow-up study[J]. BMC Cancer, 2018, 18(1):988.
